# Supplementary material for: Birhan maternal and child health cohort: a study protocol
Source: BMJ Open. 2021 Sep 28;11(9):e049692. doi: 10.1136/bmjopen-2021-049692 (PMC8480011; doi:10.1136/bmjopen-2021-049692)
Supplement: Author's manuscript [file bmjopen-2021-049692.draft_revisions.pdf]

BMJ Open is committed to open peer review. As part of this commitment we make the peer review history of every article we publish publicly available.

When an article is published we post the peer reviewers' comments and the authors' responses online. We also post the versions of the paper that were used during peer review. These are the versions that the peer review comments apply to.

The versions of the paper that follow are the versions that were submitted during the peer review process. They are not the versions of record or the final published versions. They should not be cited or distributed as the published version of this manuscript.

BMJ Open is an open access journal and the full, final, typeset and author-corrected version of record of the manuscript is available on our site with no access controls, subscription charges or pay-per-view fees (http://bmjopen.bmj.com).

If you have any questions on BMJ Open's open peer review process please email info.bmjopen@bmj.com

BMJ Open

Birhan Maternal and Child Health cohort: a study protocol

Journal:	*BMJ Open*
Manuscript ID	bmjopen-2021-049692
Article Type:	Protocol
Date Submitted by the Author:	05-Feb-2021

Complete List of Authors:	Chan, Grace; St Paul's Hospital Millennium Medical College, Department of Pediatrics and Child Health; Harvard Medical School, Harvard University T H Chan School of Public Health, Departments of Pediatrics and Epidemiology<br>Hunegnaw, Bezawit; Saint Paul's Hospital Millennium Medical College, Department of Pediatrics and Child Health<br>Van Wickle, Kimiko; Harvard University T H Chan School of Public Health, Department of Epidemiology<br>Mohammed, Yahya; Saint Paul's Hospital Millennium Medical College, HaSET<br>Hunegnaw, Mesfin; Saint Paul's Hospital Millennium Medical College, HaSET<br>Goddard, Frederick; Harvard University T H Chan School of Public Health, Department of Epidemiology<br>Tadesse, Fisseha ; Debre Birhan Referral Hospital, Department of Obstetrics and Gynecology<br>Bekele, Delayehu; Saint Paul's Hospital Millennium Medical College, Department of Obstetrics and Gynecology; Harvard University T H Chan School of Public Health, Department of Epidemiology

Keywords:	PUBLIC HEALTH, EPIDEMIOLOGY, PERINATOLOGY, Maternal medicine < OBSTETRICS, Community child health < PAEDIATRICS, NEONATOLOGY

SCHOLARONE™
Manuscripts

I, the Submitting Author has the right to grant and does grant on behalf of all authors of the Work (as defined in the below author licence), an exclusive licence and/or a non-exclusive licence for contributions from authors who are: i) UK Crown employees; ii) where BMJ has agreed a CC-BY licence shall apply, and/or iii) in accordance with the terms applicable for US Federal Government officers or employees acting as part of their official duties; on a worldwide, perpetual, irrevocable, royalty-free basis to BMJ Publishing Group Ltd ("BMJ") its licensees and where the relevant Journal is co-owned by BMJ to the co-owners of the Journal, to publish the Work in this journal and any other BMJ products and to exploit all rights, as set out in our licence.

The Submitting Author accepts and understands that any supply made under these terms is made by BMJ to the Submitting Author unless you are acting as an employee on behalf of your employer or a postgraduate student of an affiliated institution which is paying any applicable article publishing charge ("APC") for Open Access articles. Where the Submitting Author wishes to make the Work available on an Open Access basis (and intends to pay the relevant APC), the terms of reuse of such Open Access shall be governed by a Creative Commons licence – details of these licences and which Creative Commons licence will apply to this Work are set out in our licence referred to above.

Other than as permitted in any relevant BMJ Author's Self Archiving Policies, I confirm this Work has not been accepted for publication elsewhere, is not being considered for publication elsewhere and does not duplicate material already published. I confirm all authors consent to publication of this Work and authorise the granting of this licence.

For peer review only

TITLE

Birhan Maternal and Child Health cohort: a study protocol

Authors

Grace J. Chan, MD MPH PhD[1,2,3], Bezawit Mesfin Hunegnaw, MD MPH[1], Kimiko Van Wickle, BSc MSPH[2], Yahya Mohammed, BSc[4], Mesfin Hunegnaw MD MPH[4], Frederick G. B. Goddard MS PhD[2], Fisseha Tadesse MD[5], Delayehu Bekele MD MPH[2,6]

Affiliations

[1]Department of Pediatrics and Child Health, St. Paul's Hospital Millennium Medical College, Addis Ababa, Ethiopia; [2]Department of Epidemiology, Harvard T.H. Chan School of Public Health, Boston, MA, USA; [3]Division of Medicine Critical Care, Boston Children's Hospital, Department of Pediatrics, Harvard Medical School, Boston, MA, USA; [4]HaSET, St. Paul's Hospital Millennium Medical College, Addis Ababa Ethiopia; [5]Department of Obstetrics and Gynecology, Debre Birhan Referral Hospital, Debre Birhan, Ethiopia; [6]Department of Obstetrics and Gynecology, St. Paul's Hospital Millennium Medical College, Addis Ababa, Ethiopia.

Corresponding Author

Grace J Chan, MD MPH PhD

677 Huntington Ave, Kresge 913, Boston, MA 02115

grace.chan@hsph.harvard.edu

Word Count

2,670 words (excluding title page, abstract, references, figures, and tables)

Keywords

Maternal, child, health, surveillance, protocol, Ethiopia

For peer review only

ABSTRACT

Introduction
Reliable estimates on maternal and child morbidity and mortality are essential for health programs and policies. Data are needed in populations which have the highest burden of disease but also have the least evidence and research, to design and evaluate health interventions to prevent illnesses and deaths that occur worldwide each year.

Methods and analysis
The Birhan Maternal and Child Health (MCH) cohort is an open prospective pregnancy and birth cohort nested within the Birhan health and demographic surveillance system (HDSS). An estimated 2500 pregnant women are enrolled each year and followed through pregnancy, birth, and the postpartum period. Newborns are followed through two years of life to assess growth and development. Baseline medical data, signs and symptoms, laboratory test results, anthropometrics, and pregnancy and birth outcomes (stillbirth, preterm birth, low birthweight) are collected from both home and health facility visits. We will calculate the period prevalence and incidence of primary morbidity and mortality outcomes.

Ethics and Dissemination
The cohort has received ethical approval. Findings will be disseminated at scientific conferences, peer-reviewed journals, and to relevant stakeholders including the ministry of health.

Strengths and limitations of this study

- This cohort collects longitudinal data at multiple time points from pregnancy through birth and childhood in a setting where there are limited data.
- Data from this study provide estimates for birth outcomes such as stillbirths, preterm birth, and low birth weight.
- Results will inform risk profiles for maternal, neonatal, and child morbidity and mortality.
- Similar to all observational studies, there are potential confounders that are unmeasurable.
- Evidence from this study will support policies and programs to improve maternal and child health.

For peer review only

INTRODUCTION

Globally, there has been significant progress in improving maternal and child health over the past few years. However, maternal, neonatal, and child mortality remains disproportionately high in parts of the world that have limited data, research, and programs. Almost 94% of maternal mortality occurs in low-middle income country (LMIC) settings from preventable causes.[1] Sub-Saharan Africa has the highest under-five mortality.[2] There is a significant gap in our knowledge of the rates of stillbirths and where they occur. Globally, an estimated 2.6 million (uncertainty range 2.4-3.0 million) third trimester stillbirths occurred in 2015 with 98% estimated to have occurred in LMIC settings.[3 4]

In Ethiopia, the second most populous country in Africa, the maternal mortality rate is 412/100,000 live births totaling 14,000 maternal deaths each year.[5-7] Ethiopia is one of five countries which contributed to more than half of the global under-five deaths in 2018.[8] With 80,000 deaths each year, Ethiopia is one of ten countries accounting for more than half of global neonatal deaths.[9] The rate of stillbirths at a population-level in Ethiopia remains unmeasured. Rough estimates range from 9.2 stillbirths per 1000 births based on data from the 2016 Ethiopia Demographic and Health Survey (EHDS) maternal report of a pregnancy loss after seven months gestation in the last 5 years to 28 stillbirths per 1000 births in 2012 from a systematic review of predominantly hospital-based studies.[5 10 11]

Priority-setting for policies, programs and research to prevent adverse maternal and neonatal outcomes relies on accurate and timely health data.[12] Data are needed for the implementation of health interventions to prevent maternal and child deaths and morbidities. However, data on basic vital statistics is lacking in most low-income countries. In Sub-Saharan Africa, for example, fewer than ten countries have vital registration systems that produce usable data.[13]

There are significant gaps in counting every pregnancy, newborn, and child; identifying high-risk populations; and understanding causal pathways for morbidity and mortality to develop and test interventions. Many maternal and neonatal deaths are preventable with improved identification of high-risk pregnancies; treatment and management of maternal complications; and improved quality of care at birth and during the postnatal and early childhood period.[3 4] Access to timely, accurate, and dependable information is essential to improving health and health systems. To address these gaps, the Birhan Maternal and Child (MCH) cohort is designed to describe the epidemiology of maternal and childhood health outcomes and risk factors in North Shewa Zone, Amhara Region, Ethiopia. Specific objectives include:

1. To estimate the incidence of birth outcomes, including stillbirths, early neonatal mortality, preterm birth, and low birth weight.
2. To identify severe illnesses and deaths among pregnant women and children under-2 years old and identify risk factors for morbidity and mortality.
3. To build the capacity of the Ethiopian Federal Ministry of Health (FMoH) and local institutions to generate and use local evidence for action to improve maternal and child health.

METHODS AND ANALYSIS

Study design

The Birhan MCH cohort is an open prospective pregnancy and birth cohort nested within the Birhan health and demographic surveillance system (HDSS). Briefly, the Birhan HDSS includes 16 kebeles (lowest administrative unit) in two districts and as of July 2019 includes a total of 18,933 households and 77,766 people.[14] Basic health and demographic data are collected every three months through house-to-house surveillance. During these quarterly visits, data collectors conduct pregnancy surveillance among married women of childbearing age through a series of pregnancy screening questions. Urine pregnancy tests are done for women who screened positive. Pregnant women who consent to participate are enrolled in to the MCH cohort. Mothers of and

For peer review only

children under two are also enrolled into the MCH cohort. Both clinical and epidemiological data are collected at health facilities during antenatal, postnatal, and sick visits and in the community during scheduled home visits.

Study population

Pregnant women, mothers of children under two years of age, and children under-two from the Birhan catchment population are eligible for enrollment if they meet the following inclusion criteria:

- Provide informed consent.
- Enrolled in the Birhan health and demographic surveillance system. Pregnant women must have a confirmed pregnancy (urine pregnancy test, ultrasound, or other positive signs of pregnancy).

Women and children are excluded from the study if they are from:

- Semi-formed populations living in the camps e.g., military personnel and prisoners.
- Street children/orphans who have no address as they are difficult to follow
- Those who come to the area only for work during the day and have a primary household outside the catchment area

Follow-up schedule

Once enrolled, pregnant women are followed both at home and at the health facility. The home visits are conducted every three months until 32 weeks of gestation, then every two weeks. After 36 weeks, the home visits are alternated with a phone visit every week until birth. Postpartum women are followed from birth to 42 days postpartum with scheduled home visits on days 0 (birth), 6, 28, and 42 after birth. Newborns and children under-two years are followed on days 0 (birth), 6, 28 and 42 and months 6, 12, and 24. Participants who present to health facilities for antenatal care, postnatal visits, or sick visits (outpatient or admission) also have data collected by study data collectors at the health facility. Participants are censored from the study with the following events: out-migration from the catchment area, death, lost to follow-up, and at the end of the follow-up period (See Figure 1). All participants are concurrently followed on a quarterly basis by the underlying HDSS.

Data collection

Data are collected by several sources: maternal or caretaker recall, data collector assessments in the community, health worker observations at the community, health center, and hospital level. Data collected at the time of HDSS enrollment include household data on socioeconomic status, drinking water and sanitation access, flooring/wall/roof materials, number of rooms used for sleeping, place of cooking, household possessions and ownership of land. Individual (mother, father, child, grandparent) specific data on education, employment, literacy, religion, care seeking behaviors, and immunization rates are collected. Anthropometric data including weight, height, and mid-upper arm circumference (MUAC) measurements are collected for women of child-bearing age and children under-two.

Pregnant women enrolled into the cohort receive an ultrasound for gestational age dating at their earliest ANC visit. Information on the pregnancy (birth spacing, antenatal care visits) and pregnancy-related symptoms, including history of vaginal bleeding, dysuria, headache, fever, abdominal pain, and fetal movement are collected. Maternal weight and observations of clinical signs such as pallor, jaundice, and edema are collected. For newborns enrolled in the cohort within 24 hours of birth, data on labor and delivery (location of delivery, delivery method, duration of labor, complications of birth, birth weight) and immediate newborn care (breastfeeding, cord-care, bathing, skin emollients) are collected. Mothers or caretakers recall of clinical symptoms, observations of signs of illness, and neonatal weight are collected at subsequent postpartum visits. Clinical symptoms, observations of signs of illness and possible risk factors such as sick contacts, food sources, travel, and water sanitation and hygiene practices are collected at sick visits.

To ascertain the cause of death, verbal autopsies are conducted to obtain information on the symptoms, signs, and other relevant events during the illness leading to death. Three verbal autopsy questionnaires (for deaths 0-

For peer review only

28 completed days of life; deaths of children between four weeks and 11 years of age; and deaths of persons aged 12 years and above) adopted from the standard 2016 WHO VA questionnaires are used.[15] Verbal autopsies were developed to provide information on the cause of death in communities where there is limited access to health care and medical certification of causes of death. In such situations the only viable source of information on the terminal illness is from caregivers of the deceased, most often family members. Ascertaining causes of death from such information is based on the premise that VA respondents can accurately recall the details of the various symptoms and events that occurred during the period of illness prior to death, and that such information can be used to classify the cause(s) of death into diagnostic categories based on specific symptom complexes.

Electronic data collection system and data management

We use the Birhan electronic data collection system, built from Open Data Kit (ODK)[16], for longitudinal and relational data collection. ODK is a free, open-source application used to facilitate mobile data capture. ODK can be coded using SQL to facilitate data collection, transfer and storage, and authoring of the electronic questionnaires used in the data collection process. Data can be collected on a mobile device off-line. Data are uploaded daily using mobile data to the central database. Data quality checks and logic are built into the data entry tools and data system. Data are hosted on an encrypted central database on the cloud and are de-identified. The data system is developed and maintained by two data system developers. Data are managed by a team of data scientists and data managers in Stata (Version 15.1) and R/RStudio (Version 3.6.2).

Data quality

To ensure high data quality, we developed simple user-friendly questionnaires which were piloted prior to use. We recruited and trained data collectors who met a minimum level of competency through examinations. Supervisors oversee study implementation by supporting data collectors with weekly on-the site mentorship and weekly team meetings. Supervisors independently conduct home visits on a 5% random sample of households to validate data collector performance. Within the electronic data capture system, we built single field value checks, inter-field logic checks and inter-form logic checks. In case of an error at the time of data entry, pop-up warnings are triggered, prompting the user to resolve any issues prior to saving the record. Following data collection, the dataset is systematically checked for data quality issues by a team of analysts. Data quality issues are recorded, provided to data collectors to identify solutions, and then rectified in the dataset.

Primary outcomes

Primary outcomes include:

- Severe maternal morbidity and maternal mortality: unintended outcomes of the process of labor and delivery that result in significant short- or long-term consequences to a woman's health
- Stillbirth: death prior to delivery of a fetus ≥28 weeks of gestation (or >1000 grams if gestational age is unavailable)
- 7-day neonatal mortality: death of a live-born infant at 0-6 days of life
- 28-day neonatal mortality: death of a live-born infant at 0-27 days of life
- Preterm birth: delivery prior to 37 completed weeks of gestation of a live born infant
- Low birth weight: birth weight <2500 grams of live-born infant
- Place of birth (home versus facility)
- Child morbidity: incidence of diarrhea, pneumonia, febrile illnesses[17 18]
- Causes of mortality: Numbers and rates of maternal, childhood, and adult deaths and causes of death by verbal autopsy.[19 20]

Secondary outcomes include recurrence of illnesses, readmissions, and growth as measured by weight, height, MUAC, and body mass index.

Data analysis

For peer review only

For each of the primary outcomes, we will calculate the period prevalence and incidence at community and health facilities levels. To estimate period prevalence, we will sum the two-week periods over all the participants within certain age ranges as the denominator and sum the number of episodes within that two-week period as the numerator (i.e., cases over sum of person-days observed or person-days recalled). We will estimate the incidence for each outcome by taking the sum of all the person-time contributed by each person as the denominator, and the sum of all episodes over the study period as the numerator. Missing data will be assessed through quality assurance checks by field supervisors and rectified through a documented error correction system. Remaining incomplete data will be addressed through analytical approaches. We will repeat the above analyses with varying case definitions of disease severity.

In a population of 77,000 we expect approximately 2500 pregnancies per year and 4500 children under-2 years of age. Using historical data and the literature, we expect a range of outcome rates from 2.0% to 20.0% depending on outcome of interest (Table 1). The prevalence of stillbirths is estimated to be 9.2 per 1000 births in Ethiopia and 19.7 per 1000 births (around 2.0%) in Amhara.[21] The prevalence of diarrhea over a two-week period among children under-two varies by region: 31.3%[22] in Afar, 18.5% in SNNP, 16.0% in Oromia, 17.7% in Amhara, 6.8% in Tigray[23]. Given this variation, we estimate the prevalence of diarrhea over a two-week period among children under-two to be 20.0%. We expect the prevalence of acute respiratory infection over a two-week period among children under-two to be 5.0% based on preliminary data in Ethiopia.[23]

To estimate absolute precision (defined as the half-width of the 95% confidence interval) for outcomes ranging in prevalence from 2.0% to 20.0%, the exact (Clopper Pearson) distribution was used for rare outcomes (<5%) and the Wald distribution was used for common outcomes (≥5%).[24 25] A sample size of 2500 pregnant women would estimate the prevalence for a rare outcome such as stillbirth, 2.0%, with 0.6% precision (95% CI 1.4% to 2.6%) (Table 1). For more common outcomes such as preterm birth, a sample size of 2500 pregnant women would estimate a 12.0% prevalence with 1.3% precision (95% CI 10.7% to 13.3%). A sample size of 4500 children would estimate a 20.0% prevalence of diarrhea with 1.2% precision (95% CI 18.8-21.2%). A sample size of 4500 children would estimate a 5.0% prevalence of ARI with 0.5% precision (95% CI 4.5-5.5%). Period prevalence will vary based on case definitions and severity of illnesses. To examine risk factors and correlates for these outcomes, we will conduct tests of association to detect statistically significant effect sizes for risk factors and outcomes assessed in this study.

Table 1. Outcomes of interest, estimated prevalence, and precision (95% CI width) by sample size*

Outcomes	Estimated prevalence	2500	3500	4500
Stillbirth[21]	2.0%	0.6%	0.5%	0.4%
Neonatal death (28-day)[5]	3.0%	0.7%	0.6%	0.5%
Acute respiratory infections**	12.0%	1.3%	1.1%	0.9%
Diarrhea**[23]	20.0%	1.6%	1.3%	1.2%

* Precision calculated using exact distribution for <5% prevalence and Wald distribution for ≥5% prevalence
**Estimated two-week point prevalence for children under-2 years old

ETHICS AND DISSEMINATION

The study has received the following ethical approvals: St. Paul's Hospital Millennium Medical College (PM23/274), Boston Children's Hospital (P00028224) and Harvard School of Public Health (19-0991). Results will be made available to the ministry of health, regional, zonal and district health offices. Publications will follow the HaSET publications guidelines. Birhan serves as the field site for HaSET ('happiness' in Amharic)—a maternal and child health research program in Ethiopia. For further details, please visit www.hasetmch.org.

Patient and public involvement

The scope of the research questions and outcome measurements were informed by identified gaps in maternal and child health and key priorities at the Ministiry of Health including limited reliable health and demographic data and maternal and child health outcomes, that is needed to support evidence-based decision making. To build capacity on research methodologies, data analyses, and interpretation of data, fellows and trainees are involved with the cohort study. A community advisory board is engaged with the research.

Access to data

Data use is governed by the Birhan Data Access Committee (DAC) and follows Birhan's data sharing policy. All researchers who wish to access Birhan data can complete a Birhan data request form and submit it for decision by the Birhan DAC. Datasets will only be provided with de-identified data to maintain confidentiality of study participants.

ACKNOWLEDGEMENTS

We are grateful to the community of the Birhan field site, especially the mothers and children who participate. We appreciate the generous support from the Ministry of Health, Amhara Regional Health Bureau, North Shewa Zone Office, Angolela Tera and Kewet/Shewa Robit Woreda Health Offices and catchment health facilities. We thank the data collectors, supervisors, coordinators and the HaSET team for their contributions to the Birhan MCH study. St. Paul's Hospital

CONTRIBUTORS

The study concept was conceived by GC and DB. GC, DB and BH contributed with study design and implementation. MH and CB led the data collection. YM, KW, FG contributed to data management. Analysis will be performed by KM, KW, and FG. GC drafted the initial manuscript. All authors contributed to the refinement of the protocol, provided edits, and critiqued the manuscript for intellectual content.

COMPETING INTERESTS STATEMENT

None declared

FUNDING

This work has been supported by the Bill & Melinda Gates Foundation (Grant number INV-010382 and OPP1201842).

For peer review only

REFERENCES

1. Maternal mortality facts sheets: World Health Organization; 2019 [Available from: https://www.who.int/news-room/fact-sheets/detail/maternal-mortality.
2. World Health Organization. Children: improving survival and well-being September 8, 2020 [Available from: https://www.who.int/news-room/fact-sheets/detail/children-reducing-mortality#:~:text=Sub%2DSaharan%20Africa%20remains%20the,in%2013%20rate%20in%201999.
3. Yoshida S, Martines J, Lawn JE, et al. Setting research priorities to improve global newborn health and prevent stillbirths by 2025. *Journal of global health* 2016;6(1):010508. doi: 10.7189/jogh.06.010508
4. Lawn JE, Blencowe H, Waiswa P, et al. Stillbirths: rates, risk factors, and acceleration towards 2030. *The Lancet* 2016 doi: 10.1016/s0140-6736(15)00837-5
5. EDHS. Ethiopia Demographic and Health Survey Prelminary Report 2016. Addis Ababa, Ethiopia and Calverton, Maryland: Central Statistical Authority and ORC Macro. 2016
6. EDHS. Ethiopia Mini Demographic and Health Survey 2019: Key Indicators. Rockville, Maryland, USA: EPHI and ICF: Ethiopian Public Health Institute (EPHI) [Ethiopia] and ICF, 2019.
7. World Health Organization. Trends in maternal mortality 2000 to 2017: estimates by WHO, UNICEF, UNFPA, World Bank Group and the United Nations Population Division, 2019.
8. Levels & Trends in Child Mortality Report 2019, Estimates developed by the UN Inter-agency Group for Child Mortality Estimation: United Nations Children's Fund, 2019.
9. Lang T, Delarocque E, Astagneau P, et al. Hypertension during pregnancy in Africa and infants' health. A cohort study in an urban setting. *J Perinat Med* 1993;21(1):13-24. [published Online First: 1993/01/01]
10. Berhan Y, Berhan A. Perinatal mortality trends in Ethiopia. *Ethiopian journal of health sciences* 2014;24 Suppl:29-40. doi: 10.4314/ejhs.v24i0.4s [published Online First: 2014/12/10]
11. Tesema GA, Gezie LD, Nigatu SG. Trends of stillbirth among reproductive-age women in Ethiopia based on Ethiopian demographic and health surveys: a multivariate decomposition analysis. *BMC Pregnancy Childbirth* 2020;20(1):193. doi: 10.1186/s12884-020-02880-5 [published Online First: 2020/04/02]
12. Mathers C. D ea. counting the dead and What they died from: An Assessment of the global status of cause of death data. Bulletin of the World Health Organization, 2005, 83(3). 2005
13. Velkoff VA. and Setel P. Sample vital Registration using Vebal Autopsy: A new approach for collecting mortality data. International Programs Center Population Division, US Census Bureau,. 2005
14. Bekele D, Hunegnaw BM, Bekele C, et al. Cohort Profile: The Birhan Health and Demographic Surveillance System. *medRxiv* 2021:2021.01.21.21249989. doi: 10.1101/2021.01.21.21249989
15. WHO. Verbal Autopsy Standards. Ascertaining and attributing cause of death. WHO, Geneva, Switzerland.
16. Open Data kit (ODK): University of Washington's Computer Science and Engineering department; 2020 [cited 2020 August]. Available from: https://www.opendatakit.org/.
17. Breiman RF, Van Beneden CA, Farnon EC. Surveillance for respiratory infections in low- and middle-income countries: experience from the Centers for Disease Control and Prevention's Global Disease Detection International Emerging Infections Program. *J Infect Dis* 2013;208 Suppl 3:S167-72. doi: 10.1093/infdis/jit462
18. Izadnegahdar R, Cohen AL, Klugman KP, et al. Childhood pneumonia in developing countries. *The Lancet Respiratory medicine* 2013;1:574-84. doi: 10.1016/S2213-2600(13)70075-4
19. Kalter HD, Salgado R, Babille M, et al. Social autopsy for maternal and child deaths: a comprehensive literature review to examine the concept and the development of the method. *Popul Health Metr* 2011;9:45. doi: 10.1186/1478-7954-9-45 [published Online First: 2011/08/09]
20. Kalter HD, Yaroh AG, Maina A, et al. Verbal/social autopsy study helps explain the lack of decrease in neonatal mortality in Niger, 2007-2010. *Journal of global health* 2016;6(1):010604. doi: 10.7189/jogh.06.010604
21. Tesema GA, Gezie LD, Nigatu SG. Spatial distribution of stillbirth and associated factors in Ethiopia: a spatial and multilevel analysis. *BMJ Open* 2020;10(10):e034562. doi: 10.1136/bmjopen-2019-034562

For peer review only

22. Gizaw ZZ. Child feeding practices and diarrheal disease among children less than two years of age of the nomadic people in Hadaleala District, Afar Region, Northeast Ethiopia. *International breastfeeding journal* 2017;12(1):24.
23. The Last Ten Kilometers Project. Trends in reproductive, maternal, newborn and child health care practices in 115 L10K woredas: Analyses of three rounds of survey data. Addis Ababa, Ethiopia: JSI Research & Training Institute, Inc.,, 2015.
24. Clopper CJ, Pearson ES. The Use of Confidence or Fiducial Limits Illustrated in the Case of the Binomial. *Biometrika* 1934;26(4):404-13. doi: 10.2307/2331986
25. Wald A. The Fitting of Straight Lines if Both Variables are Subject to Error. *The Annals of Mathematical Statistics* 1940;11(3):284-300.

For peer review only

Figure 1: MCH Schedule and Forms

For peer review only

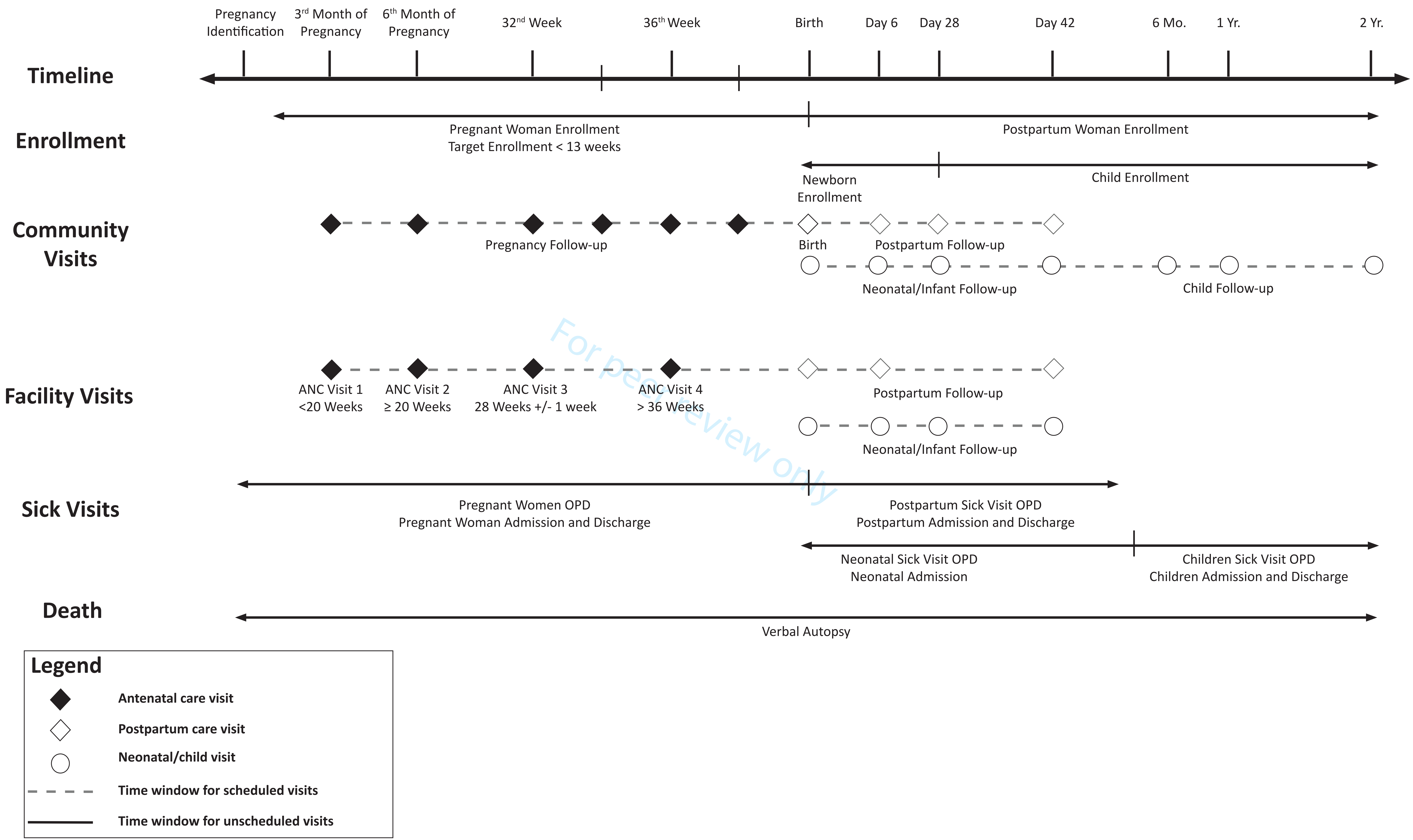

For peer review only

BMJ Open

Birhan Maternal and Child Health cohort: a study protocol

Journal:	*BMJ Open*
Manuscript ID	bmjopen-2021-049692.R1
Article Type:	Protocol
Date Submitted by the Author:	26-Jul-2021
Complete List of Authors:	Chan, Grace; St Paul's Hospital Millennium Medical College, Department of Pediatrics and Child Health; Harvard Medical School, Harvard University T H Chan School of Public Health, Departments of Pediatrics and Epidemiology<br>Hunegnaw, Bezawit; Saint Paul's Hospital Millennium Medical College, Department of Pediatrics and Child Health<br>Van Wickle, Kimiko; Harvard University T H Chan School of Public Health, Department of Epidemiology<br>Mohammed, Yahya; St Paul's Hospital Millennium Medical College, HaSET<br>Hunegnaw, Mesfin; St Paul's Hospital Millennium Medical College, HaSET<br>Bekele, Chalachew; St Paul's Hospital Millennium Medical College, HaSET<br>Goddard, Frederick; Harvard University T H Chan School of Public Health, Department of Epidemiology<br>Tadesse, Fisseha ; Debre Birhan Referral Hospital, Department of Obstetrics and Gynecology<br>Bekele, Delayehu; Saint Paul's Hospital Millennium Medical College, Department of Obstetrics and Gynecology; Harvard University T H Chan School of Public Health, Department of Epidemiology
**Primary Subject Heading**:	Global health
Secondary Subject Heading:	Epidemiology, Public health, Paediatrics, Obstetrics and gynaecology
Keywords:	PUBLIC HEALTH, EPIDEMIOLOGY, PERINATOLOGY, Maternal medicine < OBSTETRICS, Community child health < PAEDIATRICS, NEONATOLOGY

SCHOLARONE™
Manuscripts

I, the Submitting Author has the right to grant and does grant on behalf of all authors of the Work (as defined in the below author licence), an exclusive licence and/or a non-exclusive licence for contributions from authors who are: i) UK Crown employees; ii) where BMJ has agreed a CC-BY licence shall apply, and/or iii) in accordance with the terms applicable for US Federal Government officers or employees acting as part of their official duties; on a worldwide, perpetual, irrevocable, royalty-free basis to BMJ Publishing Group Ltd ("BMJ") its licensees and where the relevant Journal is co-owned by BMJ to the co-owners of the Journal, to publish the Work in this journal and any other BMJ products and to exploit all rights, as set out in our licence.

The Submitting Author accepts and understands that any supply made under these terms is made by BMJ to the Submitting Author unless you are acting as an employee on behalf of your employer or a postgraduate student of an affiliated institution which is paying any applicable article publishing charge ("APC") for Open Access articles. Where the Submitting Author wishes to make the Work available on an Open Access basis (and intends to pay the relevant APC), the terms of reuse of such Open Access shall be governed by a Creative Commons licence – details of these licences and which Creative Commons licence will apply to this Work are set out in our licence referred to above.

Other than as permitted in any relevant BMJ Author's Self Archiving Policies, I confirm this Work has not been accepted for publication elsewhere, is not being considered for publication elsewhere and does not duplicate material already published. I confirm all authors consent to publication of this Work and authorise the granting of this licence.

For peer review only

TITLE

Birhan Maternal and Child Health cohort: a study protocol

Authors

Grace J. Chan, MD MPH PhD[1,2,3], Bezawit Mesfin Hunegnaw, MD MPH[1], Kimiko Van Wickle, BSc MSPH[2], Yahya Mohammed, BSc[4], Mesfin Hunegnaw, MD MPH[4], Chalachew Bekele, MPH[4], Frederick G. B. Goddard, MS PhD[2], Fisseha Tadesse, MD[5], Delayehu Bekele, MD MPH[2,6]

Affiliations

[1]Department of Pediatrics and Child Health, St. Paul's Hospital Millennium Medical College, Addis Ababa, Ethiopia; [2]Department of Epidemiology, Harvard T.H. Chan School of Public Health, Boston, MA, USA; [3]Division of Medicine Critical Care, Boston Children's Hospital, Department of Pediatrics, Harvard Medical School, Boston, MA, USA; [4]HaSET, St. Paul's Hospital Millennium Medical College, Addis Ababa Ethiopia; [5]Department of Obstetrics and Gynecology, Debre Birhan Referral Hospital, Debre Birhan, Ethiopia; [6]Department of Obstetrics and Gynecology, St. Paul's Hospital Millennium Medical College, Addis Ababa, Ethiopia.

Corresponding Author

Grace J Chan, MD MPH PhD
677 Huntington Ave, Kresge 913, Boston, MA 02115
grace.chan@hsph.harvard.edu

Word Count

2,884 words (excluding title page, abstract, references, figures, and tables)

Keywords

Maternal, child, health, surveillance, protocol, Ethiopia

For peer review only

ABSTRACT

Introduction
Reliable estimates on maternal and child morbidity and mortality are essential for health programs and policies. Data are needed in populations which have the highest burden of disease but also have the least evidence and research, to design and evaluate health interventions to prevent illnesses and deaths that occur worldwide each year.

Methods and analysis
The Birhan Maternal and Child Health (MCH) cohort is an open prospective pregnancy and birth cohort nested within the Birhan health and demographic surveillance system (HDSS). An estimated 2500 pregnant women are enrolled each year and followed through pregnancy, birth, and the postpartum period. Newborns are followed through two years of life to assess growth and development. Baseline medical data, signs and symptoms, laboratory test results, anthropometrics, and pregnancy and birth outcomes (stillbirth, preterm birth, low birthweight) are collected from both home and health facility visits. We will calculate the period prevalence and incidence of primary morbidity and mortality outcomes.

Ethics and Dissemination
The cohort has received ethical approval. Findings will be disseminated at scientific conferences, peer-reviewed journals, and to relevant stakeholders including the ministry of health.

Strengths and limitations of this study
- This cohort collects longitudinal data at multiple time points from pregnancy through birth and childhood in a setting where there are limited data.
- The longitudinal nature of the cohort will support time to event analysis.
- The study generates accurate estimates of birth outcomes such as stillbirths, preterm birth, and low birth weight with near complete follow-up, gestational age dating with ultrasound, and digital weight measures for community, home and facility births.
- Similar to all observational studies, there are potential confounders that are unmeasurable.
- As with any longitudinal study, there is study participant attrition and missing data will be managed with imputation methods during analysis.

INTRODUCTION
Globally, there has been significant progress in improving maternal and child health over the past few years. However, maternal, neonatal, and child mortality remains disproportionately high in parts of the world that have limited data, research, and programs. Almost 94% of maternal mortality occurs in low- middle- income country (LMIC) settings from preventable causes.[1] Sub-Saharan Africa has the highest under-five mortality.[2] There is a significant gap in our knowledge of the rates of stillbirths and where they occur. An estimated 2.6 million (uncertainty range 2.4-3.0 million) third trimester stillbirths occurred in 2015 worldwide with 98% estimated to have occurred in LMIC settings.[3 4]

In Ethiopia, the second most populous country in Africa, the maternal mortality rate is 412/100,000 live births totaling 14,000 maternal deaths each year.[5-7] Ethiopia is one of five countries which contributed to more than half of the global under-five deaths in 2018.[8] With 80,000 deaths each year, Ethiopia is one of ten countries accounting for more than half of global neonatal deaths.[9] The rate of stillbirths at a population-level in Ethiopia remains unmeasured. Rough estimates range from 9.2 stillbirths per 1000 births based on data from the 2016 Ethiopia Demographic and Health Survey (EHDS) maternal report of a pregnancy loss after seven months gestation in the last 5 years to 28 stillbirths per 1000 births in 2012 from a systematic review of predominantly hospital-based studies.[10-12]

For peer review only

Priority-setting for policies, programs and research to prevent adverse maternal and neonatal outcomes relies on accurate and timely health data.[13] Data are needed for the implementation of health interventions to prevent maternal and child deaths and morbidities. However, data on basic vital statistics are lacking in most low-income countries. In Sub-Saharan Africa, for example, fewer than ten countries have vital registration systems that produce usable data.[14]

There are significant gaps in counting every pregnancy, newborn, and child; identifying high-risk populations; and understanding causal pathways for morbidity and mortality to develop and test interventions. Many maternal and neonatal deaths are preventable with improved identification of high-risk pregnancies; treatment and management of maternal complications; and improved quality of care at birth and during the postnatal and early childhood period.[3 4] Access to timely, accurate, and dependable information is essential to improving health and health systems. To address these gaps, the Birhan Maternal and Child (MCH) cohort is designed to describe the epidemiology of maternal and childhood health outcomes and risk factors in North Shewa Zone, Amhara Region, Ethiopia. Specific objectives include:

1. To estimate the incidence of birth outcomes, including stillbirths, early neonatal mortality, preterm birth, and low birth weight.
2. To identify severe illnesses and deaths among pregnant women and children under-2 years old and identify risk factors for morbidity and mortality.
3. To build the capacity of the Ethiopian Federal Ministry of Health (FMoH) and local institutions to generate and use local evidence for action to improve maternal and child health.

METHODS AND ANALYSIS

Study design

The Birhan MCH cohort is an open prospective pregnancy and birth cohort nested within the Birhan health and demographic surveillance system (HDSS). Briefly, the Birhan HDSS includes 16 kebeles (lowest administrative unit) in two districts in Amhara, Ethiopia. As of July 2019, the HDSS includes a total of 18,933 households and 77,766 people.[15] The site includes eight health facilities (three hospitals and five health centers), where all health services for pregnant women, postpartum women, and children under five are provided free of charge by the Ministry of Health. The Birhan MCH cohort started in December 2018 and will continue through 2024 with plans to continue as funding allows. Basic health and demographic data are collected every three months through house-to-house surveillance. During these quarterly visits, data collectors conduct pregnancy surveillance among married women of childbearing age through a series of pregnancy screening questions. Urine pregnancy tests are done for women who screened positive. Nonmarried women are not screened for pregnancy to respect cultural norms; nonmarried women who are visibly pregnant may be consented. Pregnant women who consent to participate are enrolled in to the MCH cohort. Mothers of and children under two are also enrolled into the MCH cohort. Both clinical and epidemiological data are collected at health facilities during antenatal, postnatal, and sick visits and in the community during scheduled home visits.

Study population

Pregnant women, mothers of children under two years of age, and children under-two from the Birhan catchment population are eligible for enrollment if they meet the following inclusion criteria:

- Provide informed consent.
- Enrolled in the Birhan health and demographic surveillance system. Pregnant women must have a confirmed pregnancy (urine pregnancy test, ultrasound, or other positive signs of pregnancy).

Women and children are excluded from the study if they are from:

- Semi-formed populations living in the camps e.g., military personnel and prisoners.
- Street children/orphans who have no address as they are difficult to follow
- Those who come to the area only for work during the day and have a primary household outside the catchment area

Follow-up schedule

Once enrolled, pregnant women are followed both at home and at the health facility. The home visits are conducted every three months until 32 weeks of gestation, then every two weeks. After 36 weeks, home visits alternate with phone visits every week until birth. Postpartum women are followed from birth to 42 days postpartum with scheduled home visits on days 0 (birth), 6, 28, and 42 after birth. Newborns and children under-two years are followed on days 0 (birth), 6, 28 and 42 and months 6, 12, and 24. Participants who present to health facilities for antenatal care, postnatal visits, or sick visits (outpatient or admission) also have data collected by study data collectors at the health facility. Participants are censored from the study with the following events: out-migration from the catchment area, death, lost to follow-up, and at the end of the follow-up period (Figure 1). In addition, all participants (including pregnant women, postpartum women, and children <2) are concurrently followed on a quarterly basis by the underlying HDSS, which includes basic health questionnaires and an under <2 year old questionnaire on child morbidity.

Data collection

Data are collected by several sources: maternal or caretaker recall, data collector assessments in the community, health worker observations at the community, health center, and hospital level. Data collected at the time of HDSS enrollment include household data on socioeconomic status, drinking water and sanitation access, flooring/wall/roof materials, number of rooms used for sleeping, place of cooking, household possessions and ownership of land. Individual (mother, father, child, grandparent) specific data on education, employment, literacy, religion, care seeking behaviors, and immunization rates are collected. Anthropometric data including weight, height, and mid-upper arm circumference (MUAC) measurements are collected for women of child-bearing age and children under-two following standardized procedures and equipment: Seca 874 digital flat scale, Seca 354 digital baby scale; Seca 417 infantometers, and tape measures. Data collectors were recruited from the local villages with at least a diploma in clinical nursing or midwifery. Supervisors have at least a master's in public health. For study purposes, data collectors received four weeks of intensive training with lectures, interactive discussions on the study protocol coupled with practical training sessions on clinical assessments and anthropometric measurements. Following training, there is onsite supervision and mentorship every week.

Pregnant women enrolled into the cohort receive an ultrasound for gestational age dating at their earliest ANC visit. Information on the pregnancy (birth spacing, antenatal care visits) and pregnancy-related symptoms, including history of vaginal bleeding, dysuria, headache, fever, abdominal pain, and fetal movement are collected. Maternal weight and observations of clinical signs such as pallor, jaundice, and edema are collected. For newborns enrolled in the cohort, data on labor and delivery (location of delivery, delivery method, duration of labor, complications of birth, birth weight) and immediate newborn care (breastfeeding, cord-care, bathing, skin emollients) are collected as soon as possible with a target of within 24 hours of birth. Mothers or caretakers recall of clinical symptoms, observations of signs of illness, and neonatal weight are collected at subsequent postpartum visits. Sick visits include outpatient clinic visits and hospital admissions. Clinical symptoms, observations of signs of illness and possible risk factors such as sick contacts, food sources, travel, and water sanitation and hygiene practices are collected at sick visits. Additionally, data on treatment modalities, referrals and vital status are collected at discharge.

To ascertain the cause of death, verbal autopsies (VA) are conducted to obtain information on the symptoms, signs, and other relevant events during the illness leading to death. Three verbal autopsy questionnaires (for deaths 0-28 completed days of life; deaths of children between four weeks and 11 years of age; and deaths of persons aged 12 years and above which includes pregnancy related questions for women of reproductive age) adopted from the standard 2016 WHO VA questionnaires are used.[16] Verbal autopsies were developed to provide information on the cause of death in communities where there is limited access to health care and medical certification of causes of death. In such situations the only viable source of information on the terminal illness is from caregivers of the deceased, most often family members. Ascertaining causes of death from such

For peer review only

information is based on the premise that VA respondents can accurately recall the details of the various symptoms and events that occurred during the period of illness prior to death, and that such information can be used to classify the cause(s) of death into diagnostic categories based on specific symptom complexes.

Electronic data collection system and data management
We use the Birhan electronic data collection system, built from Open Data Kit (ODK)[17], for longitudinal and relational data collection. ODK is a free, open-source application used to facilitate mobile data capture. ODK can be coded using SQL to facilitate data collection, transfer and storage, and authoring of the electronic questionnaires used in the data collection process. Data can be collected on a mobile device off-line. Data are uploaded daily using mobile data to the central database. Data quality checks and logic are built into the data entry tools and data system. Data are hosted on an encrypted central database on the cloud and are de-identified. The data system is developed and maintained by two data system developers. Data are managed by a team of data scientists and data managers in Stata (Version 17.0) and R/RStudio (Version 4.0.5).[18 19]

Data quality
To ensure high data quality, we developed simple user-friendly questionnaires which were piloted prior to use. We recruited and trained data collectors who met a minimum level of competency as described above and through pre-post training examinations. Supervisors oversee study implementation by supporting data collectors with weekly on-the site mentorship and weekly team meetings. Supervisors independently conduct home visits on a 5% random sample of households to validate data collector performance. Within the electronic data capture system, we built single field value checks, inter-field logic checks and inter-form logic checks. In case of an error at the time of data entry, pop-up warnings are triggered, prompting the user to resolve any issues prior to saving the record. Following data collection, the dataset is systematically checked for data quality issues by a team of analysts. Data quality issues are recorded, provided to data collectors to identify solutions, and then rectified in the dataset.

Primary outcomes
Primary outcomes include:

- Severe maternal morbidity and maternal mortality: unintended outcomes of the process of labor and delivery that result in significant short- or long-term consequences to a woman's health
- Stillbirth: death prior to delivery of a fetus ≥28 weeks of gestation (or >1000 grams if gestational age is unavailable)
- 7-day neonatal mortality: death of a live-born infant at 0-6 days of life
- 28-day neonatal mortality: death of a live-born infant at 0-27 days of life
- Preterm birth: delivery prior to 37 completed weeks of gestation of a live born infant
- Low birth weight: birth weight <2500 grams of live-born infant
- Place of birth: home or facility
- Child morbidity: incidence of diarrhea, pneumonia, febrile illnesses[20 21]
- Causes of mortality: Numbers and rates of maternal, childhood, and adult deaths and causes of death by verbal autopsy.[22 23]

Secondary outcomes include recurrence of illnesses; readmissions; and growth as measured by weight, height, MUAC, and body mass index; maternal and child immunizations, and exclusive breastfeeding.

Data analysis
For each of the primary outcomes, we will calculate the period prevalence and incidence at community and health facilities levels. To estimate period prevalence, we will sum the two-week periods over all the participants within certain age ranges as the denominator and sum the number of episodes within that two-week period as the numerator (i.e., cases over sum of person-days observed or person-days recalled). We will estimate the incidence for each outcome by taking the sum of all the person-time contributed by each person as the

For peer review only

denominator, and the sum of all episodes over the study period as the numerator. Missing data will be assessed through quality assurance checks by field supervisors and rectified through a documented error correction system. Remaining incomplete data will be addressed through analytical approaches. We will repeat the above analyses with varying case definitions of disease severity.

In a population of 77,000 we expect approximately 2500 pregnancies per year and 4500 children under-2 years of age. Using historical data and the literature, we expect a range of outcome rates from 2.0% to 20.0% depending on outcome of interest (Table 1). The prevalence of stillbirths is estimated to be 9.2 per 1000 births in Ethiopia and 19.7 per 1000 births (around 2.0%) in Amhara.[24] The prevalence of diarrhea over a two-week period among children under-two varies by region: 31.3%[25] in Afar, 18.5% in SNNP, 16.0% in Oromia, 17.7% in Amhara, 6.8% in Tigray[26]. Given this variation, we estimate the prevalence of diarrhea over a two-week period among children under-two to be 20.0%. We expect the prevalence of acute respiratory infection over a two-week period among children under-two to be 5.0% based on preliminary data in Ethiopia.[26]

To estimate absolute precision (defined as the half-width of the 95% confidence interval) for outcomes ranging in prevalence from 2.0% to 20.0%, the exact (Clopper Pearson) distribution was used for rare outcomes (<5%) and the Wald distribution was used for common outcomes (≥5%).[27 28] A sample size of 2500 pregnant women would estimate the prevalence for a rare outcome such as stillbirth, 2.0%, with 0.6% precision (95% CI 1.4% to 2.6%) (Table 1). For more common outcomes such as preterm birth, a sample size of 2500 pregnant women would estimate a 12.0% prevalence with 1.3% precision (95% CI 10.7% to 13.3%). A sample size of 4500 children would estimate a 20.0% prevalence of diarrhea with 1.2% precision (95% CI 18.8-21.2%). A sample size of 4500 children would estimate a 5.0% prevalence of ARI with 0.5% precision (95% CI 4.5-5.5%). Period prevalence will vary based on case definitions and severity of illnesses. To examine risk factors and correlates for these outcomes, we will conduct tests of association to detect statistically significant effect sizes for risk factors and outcomes assessed in this study.

Table 1. Outcomes of interest, estimated prevalence, and precision (95% CI width) by sample size*

Outcomes	Estimated prevalence	2500	3500	4500
Stillbirth[24]	2.0%	0.6%	0.5%	0.4%
Neonatal death (28-day)[5]	3.0%	0.7%	0.6%	0.5%
Acute respiratory infections**	12.0%	1.3%	1.1%	0.9%
Diarrhea**[26]	20.0%	1.6%	1.3%	1.2%

*Precision calculated using exact distribution for <5% prevalence and Wald distribution for ≥5% prevalence
**Estimated two-week point prevalence for children under-2 years old

ETHICS AND DISSEMINATION

The study has received the following ethical approvals: St. Paul's Hospital Millennium Medical College (PM23/274), Boston Children's Hospital (P00028224) and Harvard School of Public Health (19-0991). Results will be made available to the ministry of health, regional, zonal and district health offices. Birhan serves as the field site for HaSET ('happiness' in Amharic)—a maternal and child health research program in Ethiopia. For further details, please visit www.hasetmch.org. Publications will follow HaSET publications guidelines.

Patient and public involvement

The scope of the research questions and outcome measurements were informed by identified gaps in maternal and child health and key priorities at the Ministry of Health, including limited reliable health and demographic data and maternal and child health outcomes, that are needed to support evidence-based decision making. To build capacity on research methodologies, data analyses, and interpretation of data, fellows and trainees are involved with the cohort study. A community advisory board is engaged with the research.

For peer review only

Access to data

Data use is governed by the Birhan Data Access Committee (DAC) and follows Birhan's data sharing policy. All researchers who wish to access Birhan data can complete a Birhan data request form and submit it for decision by the Birhan DAC. Datasets will only be provided with de-identified data to maintain confidentiality of study participants.

ACKNOWLEDGEMENTS

We are grateful to the community of the Birhan field site, especially the mothers and children who participate. We appreciate the generous support from the Ministry of Health, St. Paul's Hospital Millennium Medical College, Amhara Regional Health Bureau, North Shewa Zone Office, Angolela Tera and Kewet/Shewa Robit Woreda Health Offices and catchment health facilities. We thank the data collectors, supervisors, coordinators and the HaSET team for their significant contributions to the Birhan MCH study.

CONTRIBUTORS

The study concept was conceived by GC and DB. GC, DB, BH, and FT contributed with study design and implementation. MH and CB led the data collection. YM, KW, FG contributed to data management. Analysis will be performed by KW and FG. GC drafted the initial manuscript. All authors contributed to the refinement of the protocol, provided edits, and critiqued the manuscript for intellectual content.

COMPETING INTERESTS STATEMENT

None declared

FUNDING

This work has been supported by the Bill & Melinda Gates Foundation (Grant number INV-010382 and OPP1201842).

For peer review only

REFERENCES

1. Maternal mortality facts sheets: World Health Organization; 2019 [Available from: https://www.who.int/news-room/fact-sheets/detail/maternal-mortality.
2. World Health Organization. Children: improving survival and well-being September 8, 2020 [Available from: https://www.who.int/news-room/fact-sheets/detail/children-reducing-mortality.
3. Yoshida S, Martines J, Lawn JE, et al. Setting research priorities to improve global newborn health and prevent stillbirths by 2025. *Journal of global health* 2016;6(1):010508. doi: 10.7189/jogh.06.010508
4. Lawn JE, Blencowe H, Waiswa P, et al. Stillbirths: rates, risk factors, and acceleration towards 2030. *The Lancet* 2016;387(10018):587-603. doi: 10.1016/s0140-6736(15)00837-5
5. Central Statistical Agency (CSA) [Ethiopia] and ICF. Ethiopia Demographic and Health Survey. Addis Ababa, Ethiopia and Rockville, Maryland, USA: CSA and ICF, 2016.
6. EDHS. Ethiopia Mini Demographic and Health Survey 2019: Key Indicators. Rockville, Maryland, USA: Ethiopian Public Health Institute (EPHI) [Ethiopia] and ICF, 2019.
7. Trends in maternal mortality 2000 to 2017: estimates by WHO, UNICEF, UNFPA, World Bank Group and the United Nations Population Division. Geneva: World Health Organization, 2019.
8. UNICEF, WHO, World Bank Group, et al. Levels and trends in child mortality 2019: estimates developed by the UN Inter-agency Group for Child Mortality Estimation, 2019.
9. UNICEF. Neonatal mortality 2020 [cited 2021 July 21]. Available from: https://data.unicef.org/topic/child-survival/neonatal-mortality/.
10. Berhan Y, Berhan A. Perinatal mortality trends in Ethiopia. *Ethiopian journal of health sciences* 2014;24 Suppl:29-40. doi: 10.4314/ejhs.v24i0.4s [published Online First: 2014/12/10]
11. Tesema GA, Gezie LD, Nigatu SG. Trends of stillbirth among reproductive-age women in Ethiopia based on Ethiopian demographic and health surveys: a multivariate decomposition analysis. *BMC Pregnancy Childbirth* 2020;20(1):193. doi: 10.1186/s12884-020-02880-5 [published Online First: 2020/04/02]
12. EDHS. Ethiopia Demographic and Health Survey Prelminary Report 2016. Addis Ababa, Ethiopia and Calverton, Maryland: Central Statistical Authority and ORC Macro. 2016
13. Mathers CD, Ma Fat D, Inoue M, et al. Counting the dead and what they died from: an assessment of the global status of cause of death data. *Bulletin of the World Health Organization: the International Journal of Public Health* 2005;83(3):171-77.
14. Velkoff V, Setel P. Sample vital registration using vebal autopsy: a new approach for collecting mortality data: International Programs Center Population Division and US Census Bureau, 2005.
15. Bekele D, Hunegnaw BM, Bekele C, et al. Cohort profile: the Birhan Health and Demographic Surveillance System. *medRxiv* 2021:2021.01.21.21249989. doi: 10.1101/2021.01.21.21249989
16. World Health Organization. Verbal autopsy standards: ascertaining and atttributing causes of death. Geneva, Switzerland: World Health Organization, 2007.
17. Open Data Kit (ODK): University of Washington's Computer Science and Engineering department; 2020 [cited 2020 August]. Available from: https://www.opendatakit.org/.
18. Stata Statistical Software: Release 17 [program]. College Station, TX: StataCorp LLC, 2021.
19. R: A language and environment for statistical computing. R Foundation for Statistical Computing [program]. Vienna, Austria, 2021.
20. Breiman RF, Van Beneden CA, Farnon EC. Surveillance for respiratory infections in low- and middle-income countries: experience from the Centers for Disease Control and Prevention's Global Disease Detection International Emerging Infections Program. *J Infect Dis* 2013;208 Suppl 3:S167-72. doi: 10.1093/infdis/jit462
21. Izadnegahdar R, Cohen AL, Klugman KP, et al. Childhood pneumonia in developing countries. *The Lancet Respiratory medicine* 2013;1:574-84. doi: 10.1016/S2213-2600(13)70075-4
22. Kalter HD, Salgado R, Babille M, et al. Social autopsy for maternal and child deaths: a comprehensive literature review to examine the concept and the development of the method. *Popul Health Metr* 2011;9:45. doi: 10.1186/1478-7954-9-45 [published Online First: 2011/08/09]

For peer review only

23. Kalter HD, Yaroh AG, Maina A, et al. Verbal/social autopsy study helps explain the lack of decrease in neonatal mortality in Niger, 2007-2010. *Journal of global health* 2016;6(1):010604. doi: 10.7189/jogh.06.010604
24. Tesema GA, Gezie LD, Nigatu SG. Spatial distribution of stillbirth and associated factors in Ethiopia: a spatial and multilevel analysis. *BMJ Open* 2020;10(10):e034562. doi: 10.1136/bmjopen-2019-034562
25. Gizaw ZZ. Child feeding practices and diarrheal disease among children less than two years of age of the nomadic people in Hadaleala District, Afar Region, Northeast Ethiopia. *International breastfeeding journal* 2017;12(1):24.
26. The Last Ten Kilometers Project. Trends in reproductive, maternal, newborn and child health care practices in 115 L10K woredas: Analyses of three rounds of survey data. Addis Ababa, Ethiopia: JSI Research & Training Institute, Inc., 2015.
27. Clopper CJ, Pearson ES. The Use of Confidence or Fiducial Limits Illustrated in the Case of the Binomial. *Biometrika* 1934;26(4):404-13. doi: 10.2307/2331986
28. Wald A. The Fitting of Straight Lines if Both Variables are Subject to Error. *The Annals of Mathematical Statistics* 1940;11(3):284-300.

For peer review only

Figure 1. Schedule of study enrollment and follow-up visits

Legend

◆ Antenatal care visit

◇ Postpartum care visit

○ Neonatal /child visit

------ Time window for scheduled visits

═══ Time window for unscheduled visits

For peer review only

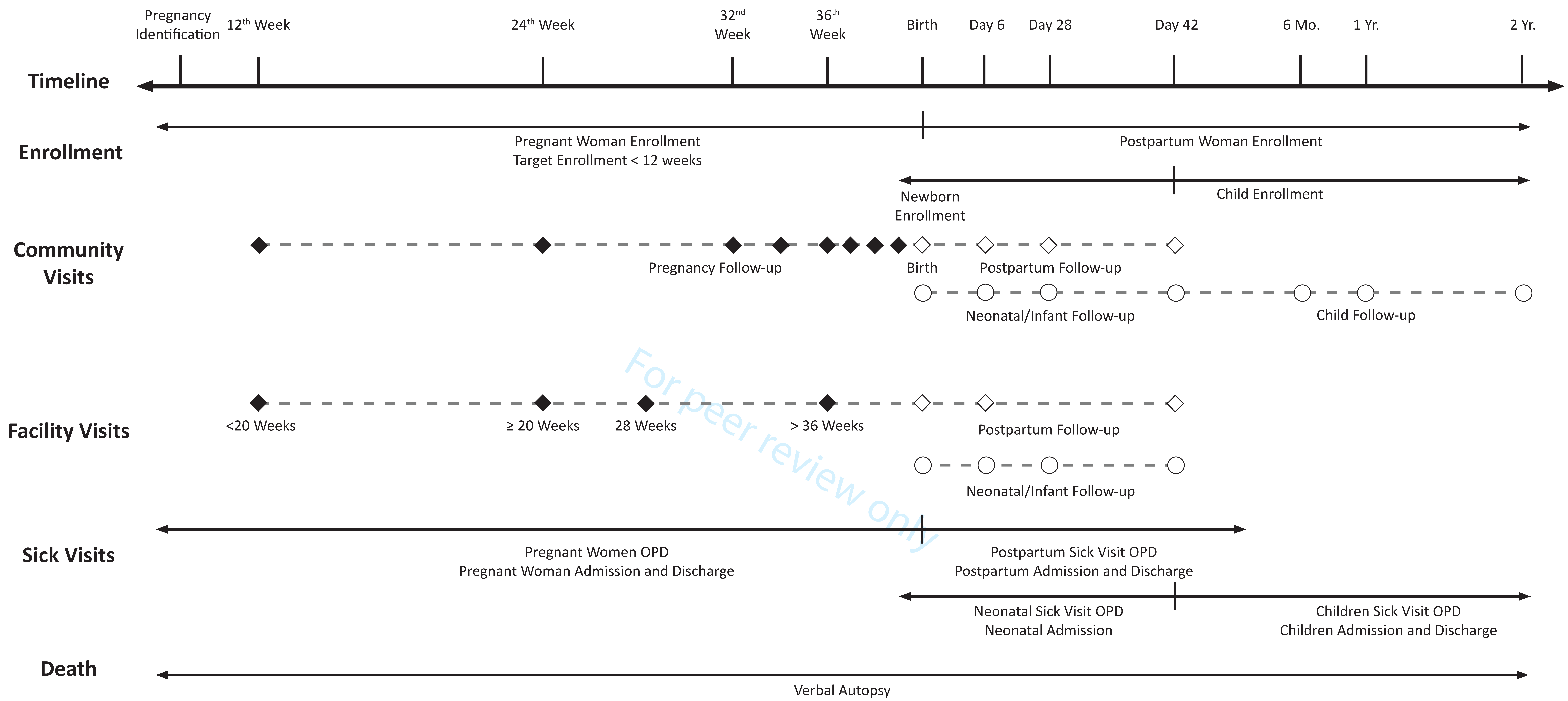

For peer review only
